# Patients’ perceptions of palliative care quality in hospice inpatient care, hospice day care, palliative units in nursing homes, and home care: a cross-sectional study

**DOI:** 10.1186/s12904-016-0152-1

**Published:** 2016-08-24

**Authors:** Tuva Sandsdalen, Vigdis Abrahamsen Grøndahl, Reidun Hov, Sevald Høye, Ingrid Rystedt, Bodil Wilde-Larsson

**Affiliations:** 1 Department of Health Studies, Faculty of Public Health, Hedmark University of Applied Sciences, Postbox 400, 2418 Elverum, Norway; 2 Department of Health Science, Faculty of Health, Science and Technology, Discipline of Nursing Science, Karlstad University, 651 88 Karlstad, Sweden; 3 Faculty of Health and Social Studies, Østfold University College, Postbox 700, 1757 Halden, Norway

**Keywords:** Palliative care, Patient perception, Quality of healthcare, Quality from the patients’ perspective questionnaire (QPP), Quality from the patients’ perspective instrument specific to palliative care (QPP-PC)

## Abstract

**Background:**

Patients’ perceptions of care quality within and across settings are important for the further development of palliative care. The aim was to investigate patients’ perceptions of palliative care quality *within* settings, including perceptions of care received and their subjective importance, and contrast palliative care quality *across* settings.

**Method:**

A cross-sectional study including 191 patients in late palliative phase (73 % response rate) admitted to hospice inpatient care, hospice day care, palliative units in nursing homes, and home care was conducted, using the Quality from the Patients’ Perspective instrument-palliative care (QPP-PC). QPP-PC comprises four dimensions and 12 factors; “medical–technical competence” (MT) (2 factors), “physical–technical conditions” (PT) (one factor), “identity–orientation approach” (ID) (4 factors), “sociocultural atmosphere” (SC) (5 factors), and three single items (S); medical care, personal hygiene and atmosphere. Data were analysed using paired-samples *t*-test and analysis of covariance while controlling for differences in patient characteristics.

**Results:**

Patients’ perceptions of care received *within* settings showed high scores for the factors and single items “honesty” (ID) and “atmosphere” (S) in all settings and low scores for “exhaustion” (MT) in three out of four settings. Patients’ perceptions of importance scored high for “medical care” (S), “honesty” (ID), “respect and empathy” (ID) and “atmosphere” (S) in all settings. No aspects of care scored low in all settings. Importance scored higher than perceptions of care received, in particular for receiving information. Patients’ perceptions of care *across* settings differed, with highest scores in hospice inpatient care for the dimensions; ID, SC, and “medical care” (S), the SC and “atmosphere” (S) for hospice day care, and “medical care” (S) for palliative units in nursing homes. There were no differences in subjective importance across settings.

**Conclusion:**

Strengths of services related to identity–orientation approach and a pleasant and safe atmosphere. Key areas for improvement related to receiving information. Perceptions of subjective importance did not differ across settings, but perceptions of care received scored higher in more care areas for hospice inpatient care, than in other settings. Further studies are needed to support these findings, to investigate why perceptions of care differ across settings and to highlight what can be learned from settings receiving high scores.

## Background

In developed countries, the number of patients in need of palliative care will continue to increase because more people are living longer, often with complex needs as a consequence of chronic illness or cancer over long periods of time [1, 2]. Modern palliative care developed from the hospice philosophy founded by Cicely Saunders and aims to improve the quality of life for patients with life-threatening illness, meet individual needs, prevent and provide relief of physical, psychosocial, and spiritual suffering, and care for patients’ families [3]. These aims should be fundamental to all palliative care services. The intention is that patients should receive high quality care [4, 5], regardless of the illness or service received [2, 3]. Fulfilment of these aims will challenge healthcare systems and services in developing palliative care to ensure good quality for patients in the future [1, 2, 6–8].

Patients’ perspectives about their care have been highlighted as being of the utmost importance in the evaluation and further development of quality in healthcare, in general [4, 9–12], and palliative care, in particular [8, 13, 14]. However, in palliative care research, studies with patients’ perspectives on care are still rare and often create methodological and ethical challenges due to e.g. the vulnerability of the patients and difficulties obtaining informed consent [15]. Family members are therefore often used as proxies to investigate patients’ views about their care. An advantage of using relatives as proxies is the opportunity to also include the views of patients in late palliative phase, who may be unable to communicate their perceptions of care quality. However, by using family members as proxies, uncertainties arise about the congruence between patient and proxy perceptions. Even if previous studies have shown that relatives and patients have mainly congruent views about the care, agreement has been poorer related to the subjective aspects of patients’ experience, such as pain, anxiety, depression and perceptions of importance of care aspects [16–18], especially when patients and relatives do not have daily contact [18]. According to the person-centred approach, the patients’ own experience, preferences, needs and values are the base upon which high quality care is built [19, 20] . This underpins the importance of including the patients’ being in the palliative phases of their illness, while addressing the ethical and methodological challenges, to gain first-hand information of the palliative care experience.

Palliative care comprises different types of services, e.g. home care, day care and inpatient services; however, the structure of these services may differ from one country to another [21]. Palliative care services may be classified as specialized (exclusively providing palliative care) and non-specialized services (occasionally providing palliative care) [1]. In Norway, palliative care is provided by a public healthcare system in primary or community care and specialist healthcare contexts (tertiary and secondary care) [22, 23]. Community care and specialist healthcare provide non-specialized palliative care (general palliative care) as an integrated part of the services, in addition specialized palliative care services are offered. Community care is responsible for and serves patients both in nursing homes and at home, via home care or their general practitioner [22]. Specialized palliative care is provided by palliative units or beds in nursing homes and cancer nurses/coordinators in the community [8]. The State is responsible for patients in specialist healthcare and serves patients in hospitals and specialist services [22]. Specialized palliative care in the specialists healthcare system is organized through palliative centres, palliative units in nursing homes and palliative care teams [8]. As part of the palliative care provided, dedicated hospice care services are specialized palliative care services using “Hospice” by name, indicating special dedication to the hospice philosophy and values [8]. Hospice services are organised within community care as palliative units in nursing homes and within the specialist healthcare system as palliative care units, palliative care teams or day care centres led by the hospitals.

Palliative care has been criticized for being developed to include mainly patients with cancer. Consequently, the care for patients with other life-threatening illnesses does not equal the care for patients with cancer [24–26]. This is also the case for palliative care research, in which more studies are needed investigating care quality from the perspectives of patients with life-threatening illnesses other than cancer [14]. When investigating patients’ perceptions of their care, it is important to include patients who receive care from various care contexts, both specialized and non-specialized, and from services provided for patients with cancer as well as for patients with other life-threatening illnesses.

In the present study quality of palliative care is based on a model from Wilde et al. [9] in which quality of care is formed through patient norms, expectations, and experience, and by the encounter with a care structure. It is therefore important, when measuring quality of care, to include both patients’ perceptions of the actual care received and how important the various care aspects are to them [27]. The advantage of including both these angles is that areas for improvement may appear to be in line with what patients perceive as most important.

Previously patients’ perceptions of important aspects of palliative care have been found to be, for example; a focus on living a meaningful life, experiencing trust, compassionate and respectful care; participating in effective communication and shared decision-making [14], and; receiving help to minimize the burden [28]. It also involves; being cared for in a safe, comfortable environment [14, 28, 29], and having organization of care that ensures access whenever needed and an experience of care that has continuity, and is well coordinated and planned [14]. Several studies that have investigated patients’ perceptions of the care received by patients being in the palliative phase [24, 25, 30–37], but studies comprising patients’ perceptions of important aspects of care, with the care actually received, are sparse. Four studies, of which three papers based on the same study from Canada, have highlighted areas for improvement in palliative care including: symptom relief [38–40], psychological and spiritual support, enhanced relationship with physicians [41], participation in decision-making [40, 41], honest communication [38, 41], planning, cooperation and continuity of care [38–41], reduced family burden [38, 39], and not being kept alive when there was little hope of a meaningful recovery [39]. Based on these four articles, further studies investigating care quality from these two angles; (a) the perceptions of care received and (b) the importance of the care aspects, are recommended.

Investigation of patients’ perceptions of care quality *within* different settings of palliative care is important for the identification of areas for improvement. Furthermore it is important to identify the strengths of each of these settings, thereby guiding further development of specific settings. The research mentioned above has included and compared patients’ views about important aspects of care and the care received [38, 39, 41], but not within different contexts of care. Other studies have investigated care quality within specific settings, e.g. advanced home care, nursing homes, hospitals, and day hospices [18, 32, 33, 42]. In these studies, there has been no comparison of patients’ perceptions of subjective importance and actual care received. In Norway, a recent report stated that there was a lack of knowledge about patients’ perceptions of quality in palliative care services, especially within community care services, including home care, palliative units in nursing homes, and hospice services [8]. Such knowledge could highlight whether patients’ perceptions of care received and preferences vary *across* settings, and build up knowledge for further research to explore what can be learned from settings that deliver high-quality care [43]. A few studies have previously compared perceptions of care quality across contexts of palliative care, investigating differences between hospice and hospital care [44] and across home care, long-term care, hospital, and hospital-based palliative care units [6], from relatives’ perspectives. The results of the two studies differed. Addington-Hall et al. [44] found that hospices gained favourable results with regard, for example, to pain control, communication, personal care, and dignity, whereas Burge et al. [6] found that none of the included contexts differed exceptionally. Therefore further studies investigating care quality across settings are important, especially those that include the patients’ perspectives.

There seems to be a need for more studies investigating patients’ perceptions of care quality from both the perspective of subjective importance and the care received *within* the different settings of palliative care, especially from the hospice, palliative units in nursing homes, and non-specialized home-care settings. Studies that have compared perceptions of care quality *across* settings are based upon relatives’ perspectives. More studies are needed to investigate patient perceptions of care quality across settings of palliative care.

The aim of the present study was therefore to investigate (1) patients’ perceptions of palliative care quality *within* settings (hospice inpatient care, hospice day-care, palliative units in nursing homes, and home care), including perceptions of care received and their subjective importance, and (2) contrast patients’ perceptions of palliative care quality *across* settings.

## Methods

### Design

The present paper is part of and analyses data from a larger cross-sectional study, using a questionnaire that measured patients’ perceptions of quality of palliative care. A paper from this study has previously been published [40].

### Setting

Participants in the present study were recruited from two inpatient hospices (HICs) (number of beds in total: 23), two hospice day-care centres (HDs) (number of patients per week in total: 65), two palliative units in nursing homes which were specialized in palliative care (PUNHs) (number of beds in total: 12), and two home-care districts (HCs) (approximately 1700 patients per year in total). The settings represented rural and urban locations in the eastern part of Norway. The hospices’ inpatient and day-care settings, and the palliative care units in the nursing homes, may be characterized as specializing in palliative care, with the setting providing exclusively palliative care [1]. The home-care settings may be characterized as non-specialized palliative care services, in which personnel care for all patients in need of homecare not exclusive patients in palliative phase. Both home-care districts did offer specialized trained personnel, such as palliative care teams and cancer nurses, to patients in a palliative phase. One of the hospices, the two palliative units in both nursing homes and the two home-care districts, were part of community care.

### Participants

As there is no consensus on the definition of this population group [45], it is important to describe the patient sample recruited [14]. In the present study, patients were defined as being in a late palliative phase [46, 47] if they were in an advanced phase of their illness, with a 1-year life expectancy [48] and experience of palliative care services [45] (specialized or non-specialized). Patients who received palliative care from the settings mentioned above were consecutively recruited and invited to participate in the study when they met the following inclusion criteria: (1) adult (≥18 years), (2) no cognitive impairment (which was judged by the registered nurse selected as responsible for recruiting patients in each site [RRN]), (3) understand Norwegian, (4) received assistance from the services for at least 3 days, and (5) has an advanced, life-threatening illness in a late palliative phase (malignant or non-malignant) (judged by the RRN), guided by the nurse’s negative response to the question: “Would you be surprised if this patient died within the next year?” [48]. For patients admitted to non- specialized palliative care service, not exclusively for patients being in late palliative phase, patients’ medical and care records were searched for documentation to indicate that the patient was in a late palliative phase, e.g. phrases like “advanced cancer/illness” or documentation of visits by palliative care team. This strategy was to aid the RRN’s decision when recruiting patients. Also included was that patients should be aware of being in a palliative phase and receiving palliative care (judged by the RRN). The patients’ physicians and one of the researchers (TS) were consulted when uncertainties arose about the inclusion criteria.

### Instruments

Patients’ perceptions of care were measured using the *Quality from the Patients’ Perspective instrument specific to palliative care (QPP-PC)* [40]. The QPP-PC is based on the theoretical foundation of the validated general instrument QPP [9, 27, 49, 50]. The QPP-PC has been psychometrically evaluated [40]. This instrument has the aim of measuring palliative care quality from the perspective of patients with various life-threatening illnesses who get help from different services. The 52-item QPP-PC instrument comprises four dimensions of quality: “medical–technical competence of the caregiver” (MT) (2 factors and 10 items), “physical–technical conditions of the care organization” (PT) (one factor and three items), “Identity–orientation approach” (ID) (4 factors and 20 items), “sociocultural atmosphere” (SC) (5 factors and 17 items), and three single items about; medical care, personal hygiene and atmosphere, representing quality of care. A description of QPP-PC has previously been published [40].

Each item of QPP-PC was answered in two ways: how the patients actually experience the care received (perceived reality – PR scale) and how important each aspect of care was to them (subjective importance – SI scale). The PR of the quality of care was measured by items related to the sentence: “This is what I experience …” (e.g. the best possible help for my pain). The items measuring the SI of care aspects were related to the sentence: “This is how important this is to me …” (e.g. the best possible help for my pain). A 4-point Likert scale, ranging from 1 (do not agree at all) to 4 (fully agree), was used for the PR and SI scales: 1 (of little or no importance) to 4 (of the very highest importance). A non-applicable response was available for both responses. For the QPP-PC dimensions and factors, a mean value was calculated based on the individual participant’s response to the items in the respective dimension or factor. In the present study Cronbach’s α for the QPP-PC showed α values >0.7 for most dimensions (0.88–0.94) and factors (0.77–0.97), except for the PT dimension where α levels were 0.44 for the PR scale and 0.65 for the SI scale and for the factors “access to help, food, and equipment” (PR 0.44, SI 0.65), “relatives and friends” (PR 0.66, SI 0.79), and “continuity” (PR 0.53, SI 0.55).

Data about *participants’ characteristics* comprised age, gender, diagnosis, time in care, education, language/ethnicity, living conditions, contact with family and friends, and religious affiliations (11 items). In addition, patient characteristics about health-related quality of life, physiological wellbeing, and sense of coherence were measured as described below.

Data on health-related quality of life were collected, using the *EQ VAS* from the EQ-5D-3 L questionnaire from the EUROQOL group [51, 52] (one item). This questionnaire is a standardized generic measure of health, designed for self-completion by respondents [53] and has been validated [54] and used by patients in various countries and settings, including palliative care. The EQ VAS measures the respondent’s self-rated health on a vertical, visual analogue scale where the endpoints are labelled “best imaginable health state” and “worst imaginable health state”.

*Physiological well-being* was measured by one item from the QPP questionnaire, related to the sentence: “I feel that my physiological well-being is…”, using a 5-point scale ranging from 1 (“very poor”) to 5 (“very good”) [27].

*The Sense of Coherence (SOC) scale* [55, 56] is a validated scale to measure patients’ life orientation in terms of how people manage stressful situations and staying well. It comprises questions about comprehensibility, manageability, and meaningfulness. In the present study the 13-item version with a 7-point scale, translated to Norwegian, was used [57]. The SOC index was calculated by adding the score from each item, ranging from 13 to 91. High scores represent a strong SOC. Cronbach’s α was 0.78 in the present study.

### Procedure

Data were collected by the instruments described above from November 2013 to December 2014. In each ward or home-care district, the RRN was responsible for screening patients and asking them to participate in the study. In addition, the RRN provided information about the study and instructions on how to fill out the questionnaire. The participants were told that they could use the time they needed to fill out the questionnaire and instructed to return the completed questionnaire in a sealed envelope. These envelopes were stored in a box or on a shelf in the RRN’s office until the researcher collected them. Patients who needed help filling out the questionnaire were offered to be interviewed by one of the researchers (TS). The interviews were conducted in the respondents’ room, a private room in the ward or in their homes. The interviewer read each question in the questionnaire aloud to the respondents. To make it easier for some patients, the response scales were enlarged on a separate sheet of paper. The patients pointed at the appropriate response category or answered verbally, whereby the researcher then wrote their responses on the scales following each question. Of the 191 participating patients, 54 received help filling out the questionnaire. Of these, 7 were from hospice inpatient care, 8 from hospice day care, 22 from palliative units in nursing homes, and 17 from home care.

### Analysis

IBM SPSS Statistics Data Editor Software [58], version 23, was used to analyse the data. Patients’ characteristics, psychological wellbeing, health-related quality of life, and sense of coherence were examined using descriptive statistics. Differences in patient characteristics in the four settings were examined using Pearson’s *χ*^2^ test for independence and analysis of variance (ANOVA), followed by Tukey’s honest significant difference test for post-hoc comparison, as appropriate [58]. Paired-samples *t*-tests were used to explore differences in patients’ perceptions of care quality including: PR and SI scales in each of the four settings – hospice inpatient care, hospice day care, palliative units in nursing homes, and home care [58].

Analysis of covariance (ANCOVA) [58, 59] was performed for each QPP-PC dimension and single items for the PR and SI scales, to compare means of patients’ perceptions of the care quality across the four settings (hospice inpatient care, hospice day care, palliative units in nursing homes, and home care) while controlling for differences in age, levels of education (primary/high school, university), and number of diagnoses (one diagnosis and two or more diagnoses) (main effect). Two-way interactions between these variables were also assessed, and statistically significant interactions were included in the analysis. Significant *F*-levels of ANCOVA were followed by Bonferroni’s test for post-hoc comparison to analyse differences in the adjusted means for the QPP-PC dimensions and single items (PR and SI scales) across the four settings. Preliminary analyses were conducted to ensure no violation of the assumptions made by use of the ANCOVA [59].

The dependent variables represented the four dimensions (MT, PT, ID, and SC) and single items (medical care, personal hygiene, and atmosphere) of the QPP-PC for both the PR and the SI scales. The continuing variable “age” was used as a covariate. The independent variables comprised the grouping variables representing the four settings and the variables that showed statistically significant differences in patient characteristics (displayed in Table 1): educational level (*P* = 0.01) and number of diagnoses (*P* = 0.01). The patient characteristics “type of diagnosis” and “time in care”, which also showed statistically significant differences between the groups, were not included in the analysis model. “Type of diagnosis” was not included due to the lack of participants with non-malignant diagnoses in hospice inpatient care. “Time in care” was not included because the differences between settings were expected to systematically vary as a result of the way in which the different services were organized. Hospice inpatient care and palliative units in nursing homes were organized as short-term services, whereas home care and hospice day care may be considered as long-term services. This made it difficult to assess the unique effect of care quality.

**Table 1:** Patient characteristics by settings of care (*n* = 191)

Characteristics	Settings of care					
		Hospice inpatient (*n* = 72)	Hospice day care (*n* = 51)	Palliative units in nursing homes (*n* = 30)	Home care (*n* = 38)	*P-value*
Age, mean score (SD)		63.81 (11.25)	62.88 (10.33)	70.00 (10.65)	74.79 (10.65)	<0.001/<0.001*
Range		41–86	42–83	50–88	55–94	
Missing	7					
Gender, *n* (%)						0.356
Female		35 (50.0)	33 (66.0)	17 (56.7)	23 (60.5)	
Male		35 (50.0)	17 (34.0)	13 (43.3)	15 (39.5)	
Missing	3					
Education, *n* (%)						<0.002
Primary school or high school (or equivalent)		34 (47.9)	33 (66.0)	23 (79.3)	29 (80.6)	
University/university college		37 (52.1)	17 (34.0)	6 (20.7)	7 (19.4)	
Missing	5					
First language^a^, *n* (%)						0.335
Norwegian		64 (88.9)	45 (90.0)	30 (100)	38 (100)	
Sami		0	0	0	0	
Other Nordic language		3 (4.2)	1 (2.0)	0	0	
Other European language		4 (5.6)	4 (8.0)	0	0	
Non-European language		1 (1.4)	0	0	0	
Missing	1					
Living conditions, *n* (%)						0.396
Living alone		34 (47.2)	30 (60.0)	17 (56.7)	16 (42.1)	
Living with a partner		29 (40.3)	15 (30.0)	10 (33.3)	20 (52.6)	
Living with children aged <18 years		7 (9.7)	2 (4.0)	1 (3.3)	1 (2.6)	
Living with others		2 (2.8)	3 (6.0)	2 (6.7)	1 (2.6)	
Missing	1					
The amount of contact with family or friends, *n*(%)						0.250
Daily		47 (66.2)	22 (44.0)	20 (66.7)	21 (55.3)	
Several times a week		19 (26.8)	22 (44.0)	8 (26.7)	15 (39.5)	
Once a week to once a month		5 (7.0)	6 (12.0)	2 (6.7)	2 (5.3)	
Less than once a month		0	0	0	0	
No contact with family or friends		0	0	0	0	
Missing	2					
The sufficiency of contact with family or friends, *n*(%)						0.675
Too often		2 (2.9)	1 (2.0)	2 (6.7)	0	
Sufficient		61 (87.1)	43 (86.0)	23 (76.7)	33 (86.8)	
Too seldom		7 (10.0)	6 (12.0)	5 (16.7)	5 (13.2)	
Missing	3					
Number of diagnoses, *n* (%)						<0.001
One diagnosis		61 (84.7)	36 (72.0)	11 (36.7)	24 (63.2)	
Two or more diagnoses		11 (15.3)	14 (28.0)	19 (63.3)	14 (36.8)	
Missing	1					
Type of diagnosis^a^, *n* (%)						<0.001
Malign diagnoses (cancer)		69 (95.8)	38 (76.0)	17 (56.7)	20 (52.6)	
Non-malign diagnoses (e.g. COPD, HF, MS, ALS, Parkinson’s disease)		0 (0)	8 (16.0)	8 (26.7)	15 (39.5)	
Mixed malign and non-malign diagnosis		3 (4.2)	4 (8.0)	5 (16.7)	3 (7.9)	
Missing	1					
Time in care (days), n (%)						<0.001
3–7		23 (34.3)	3 (6.3)	5 (20.7)	0	
8–30		25 (37.3)	7 (14.6)	18 (62.1)	2 (5.7)	
31–182 days (1–6 months)		9 (13.4)	23 (47.9)	3 (10.3)	13 (37.1)	
> 183 (>6 months)		10 (14.9)	15 (31.3)	2 (6.0)	20 (57.1)	
Missing	12					
Religious affiliation, *n* (%)						0.345
No		30 (45.5)	24 (52.2)	19 (65.5)	20 (54.1)	
Yes		36 (54.4)	22 (47.8)	10 (34.5)	17 (45.9)	
Missing	13					
Sense of coherence (SocTotal), mean score (SD)		62.56 (11.68)	61.50 (10.00)	60.77 (10.18)	65.41 (12.18)	0.407
Range		29–91	31–84	45–81	42–87	
Missing	44					
Physiological wellbeing, mean score (SD)		3.56 (0.93)	3.62 (0.83)	3.23 (1.03)	3.48 (0.96)	0.356
Missing	21					
Health-related quality of life (EQ VAS), mean score(SD)		47.95 (22.03)	50.49 (21.08)	42.69 (17.16)	48.19 (18.52)	0.480
Range		5–90	0–90	10–85	5–90	
Missing	25					

*ALS* amyotrophic lateral sclerosis, *COPD* chronic obstructive pulmonary disorder, *HF* heart failure, *MS* multiple sclerosis

^a^For descriptive purpose only. The numbers of participants in the subgroups are too few to be included in further analysis

*P* value refers to differences between patients in the four settings measured by one-way between-group ANOVA or Pearson’s *χ* ^2^ for independence, as appropriate. Statistical significance was assumed at the *P* <0.025 level

*P value refers to differences between patients within the four settings, measured with Tukey honest significant difference post-hoc comparison. Mean age for hospice inpatient and hospice day care was significantly different from that for home care (<0.001/<0.001)

Cronbach’s α was performed, for the QPP-PC, at dimension and factor levels of both subscales (PR and SI) and, for the SOC instrument, and is presented in the description of each instrument. Values >0.7 were regarded as desirable [58].

Non-response analysis was performed using independent-samples *t*-test and χ^2^ test for independence, as appropriate.

To avoid type 1 error, the statistical significance level for all the analyses was reduced to *P* <0.025, in addition conservative post-hoc tests for the ANCOVA analysis were chosen, as recommended by Tabachnick and Fidell [59].

## Results

### Patient characteristics

Of the 262 patients asked to participate, 191 returned the questionnaire (response rate = 73 %). Of the participants, 57 % were female, most were elderly, with a mean age of 67 years (standard deviation [SD] = 11.62, range: 41–94 years), had cancer (76 %), were living alone (51 %), and had a medium-to-high level (high school or university) of education (75 %). Patients who did not respond (*n* = 71) did not differ significantly from patients who responded about age (*P* = 0.569) or gender (*P* = 0.117). Of the participating patients, 72 (37 %) were recruited from hospice inpatient care, 51 (27 %) from hospice day care, 30 (16 %) from palliative units in nursing homes, and 38 (20 %) from home care.

Patient characteristics for the four settings are presented in Table 1. Participants differed significantly by location of care with respect to age, education, type of diagnosis, number of diagnoses, and time in care, but did not differ with regard to their sense of coherence, psychological wellbeing, or health-related quality of life. Patients in hospice inpatient care and hospice day care were significantly younger than those in home care. A higher proportion of patients with university education were admitted to hospice inpatient care than to hospice day care, palliative units in nursing homes, and home care. Hospice inpatient and day care had higher proportions of patients with cancer and patients with only one diagnosis than palliative units in nursing homes and home care. Length of experience with the services was shorter for patients in hospice inpatient care and palliative units in nursing homes than for patients in hospice day care and home care.

### Patients’ perceptions of quality of their care *within* settings

Table 2 presents mean values for the QPP-PC dimensions, then factors and single items within the four settings. The results are presented separately for each setting, by the levels of PR (perceived reality) and SI (subjective importance) that patients scored highest (≥3.55) and lowest (<3.00), and then by comparing the PR and SI scores.

**Table 2:** Patients’ perceptions of care received and subjective importance *within* settings, by dimensions, factors, and single items

	Hospice (*n* = 72)			Hospice day care (*n* = 51)			Palliative units in nursing homes *(n* = 30)			Home care (*n* = 38)		
Dimension/factor/single item	Perceived reality (PR)	Subjective importance (SI)		Perceived reality (PR)	Subjective importance (SI)		Perceived reality (PR)	Subjective importance (SI)		Perceived reality (PR)	Subjective importance (SI)	
	Mean (SD)	Mean (SD)	*P*	Mean (SD)	Mean (SD)	*P*	Mean (SD)	Mean (SD)	*P*	Mean (SD)	Mean (SD)	*P*
Medical–technical competence	3.21(0.64)	3.20(0.61)	0.840	3.05(0.65)	3.15(0.69)	0.255	3.05(0.74)	3.27 (0.55)	0.072	2.73 (0.75)	3.08(0.59)	0.022
Symptom relief	3.24(0.69)	3.25(0.61)	0.927	3.07(0.68)	3.15(0.72)	0.325	3.21(0.71)	3.39 (0.48)	0.151	2.83 (0.80)	3.20(0.60)	0.011
Exhaustion	3.14(0.81)	3.08(0.78)	0.466	2.93(0.80)	3.11(0.79)	0.226	2.74(1.13)	2.98 (0.94)	0.206	2.38 (1.01)	2.78(0.92)	0.103
Medical care (single item)	3.78(0.51)	3.77(0.52)	0.810	3.61(0.61)	3.82(0.49)	0.011	3.43(0.97)	3.63 (0.57)	0.161	3.24 (0.85)	3.71(0.57)	0.001
Personal hygiene (single item)	3.57(0.62)	3.47(0.70)	0.224	3.28(0.90)	3.44(0.57)	0.454	3.63(0.69)	3.56 (0.64)	0.425	3.35 (0.71)	3.48(0.59)	0.377
Physical–technical condition	3.57(0.49)	3.42(0.61)	0.046	3.43(0.70)	3.53(0.46)	0.284	3.57(0.55)	3.61 (0.49)	0.673	3.40 (0.63)	3.47(0.59)	0.566
Access to help, food, and equipment	3.57(0.49)	3.42(0.61)	0.046	3.43(0.70)	3.53(0.46)	0.284	3.57(0.55)	3.61 (0.49)	0.673	3.40 (0.63)	3.47(0.59)	0.566
Identity-oriented approach	3.51(0.45)	3.52(0.50)	0.815	3.45(0.41)	3.60(0.37)	0.009	3.09(0.52)	3.37 (0.44)	0.006	3.12 (0.54)	3.43(0.52)	<0.001
Information	3.45(0.54)	3.46(0.54)	0.802	3.11(0.67)	3.46(0.51)	<0.001	2.60(0.75)	3.12 (0.63)	<0.001	2.81 (0.74)	3.32(0.63)	<0.001
Honesty	3.69(0.51)	3.77(0.39)	0.102	3.77(0.41)	3.70(0.49)	0.406	3.83(0.35)	3.68 (0.46)	0.097	3.75 (0.46)	3.59(0.43)	0.116
Respect and empathy	3.70(0.42)	3.59(0.57)	0.019	3.79(0.30)	3.78(0.37)	0.710	3.53(0.46)	3.65 (0.50)	0.319	3.51 (0.42)	3.63(0.48)	0.043
Participation	3.20(0.76)	3.46(0.65)	0.001	3.30(0.69)	3.41(0.64)	0.214	2.83(1.05)	2.97 (0.93)	0.461	2.89 (0.86)	3.18(0.83)	0.080
Sociocultural atmosphere	3.49(0.43)	3.37(0.55)	0.018	3.40(0.47)	3.47(0.38)	0.255	3.29(0.52)	3.33 (0.53)	0.675	3.02 (0.62)	3.41(0.51)	<0.001
Meaningfulness	3.53(0.67)	3.37(0.82)	0.032	3.58(0.53)	3.54(0.61)	0.559	3.53(0.69)	3.53 (0.77)	1.000	3.11 (0.78)	3.61(0.53)	<0.001
Spiritual and existential	3.15(0.90)	3.02(0.91)	0.144	3.20(0.79)	3.12(0.96)	0.527	2.46(1.16)	2.51 (1.27)	0.814	2.68 (0.86)	3.09(1.20)	0.063
Relatives and friends	3.65(0.42)	3.52(0.57)	0.045	3.51(0.68)	3.68(0.47)	0.086	3.68(0.48)	3.68 (0.49)	1.000	3.45 (0.61)	3.65(0.41)	0.170
Continuity	3.40(0.54)	3.35(0.64)	0.487	3.25(0.73)	3.33(0.68)	0.456	2.93(0.65)	3.08 (0.59)	0.345	2.74 (0.82)	3.21(0.72)	0.012
Planning and cooperation	3.61(0.46)	3.58(0.53)	0.616	3.47(0.60)	3.67(0.42)	0.013	3.45(0.69)	3.44 (0.69)	0.939	3.16 (0.84)	3.51(0.64)	0.005
Pleasant and safe atmosphere (single item)	3.81(0.43)	3.75(0.48)	0.289	4.00(0.00)	3.82(0.39)	0.012	3.96(0.20)	3.85 (0.37)	0.083	–	–	–

*P* values refer to differences in paired-samples *t*-tests. Statistical significance was assumed at the *P* <0.025 level

### Hospice inpatient care

The highest levels of PR were experienced by patients with regard to the PT dimension, and for five factors – “access to help, food, and equipment”, “honesty”, “respect and empathy”, “relatives and friends”, “planning and cooperation” – and three single items – “medical care,” “personal hygiene”, and “pleasant and safe atmosphere”. For the PR scale, no dimensions, factors, or single items received scores <3.00.

The highest levels on the SI scale were scored for three factors – “honesty”, “respect and empathy”, and “planning and cooperation” – and two single items – “medical care” and “pleasant and safe atmosphere”. For the SI scale, no dimensions, factors, or single items received scores <3.00.

When comparing patients’ experiences with their care (PR) and how important they perceived this care (SI) to be, significant differences appeared at the dimension level for SC. For this dimension PR were scored higher than SI. At the factor level significant differences appeared in the factors “respect and empathy”, in which PR was scored higher than SI, and “participation”, in which PR was scored lower than SI. No other statistically significant difference appeared for the single items.

### Hospice day care

The highest levels of PR were scored for two factors – “honesty” and “respect and empathy” – and two single items – “medical care” and “pleasant and safe atmosphere”. The lowest PR scores were shown for the factor “exhaustion”.

For the SI scale, the highest levels were present in the ID dimension and for four factors – “honesty”, “respect and empathy”, “relatives and friends”, and “planning and cooperation” – and two single items – “medical care” and “pleasant and safe atmosphere”. For the SI scale, no dimensions, factors, or single items received scores <3.00.

Statistically significant differences between the PR and the SI scales were shown for the ID dimension and for the factor “information”, for which SI scored higher than PR. For the factor “planning and cooperation” and the single item about medical care, SI scored significantly higher than PR. For the item about atmosphere PR scored significantly higher than SI.

### Palliative units in nursing homes

For the PR scale, the highest scores were received for the PT dimension. The two factors – “honesty” and “relatives and friends” – and two single items –“personal hygiene” and “pleasant and safe atmosphere” – received the highest scores. The lowest PR scored was shown for five factors: “exhaustion”, “information”, “participation”, “spiritual and existential”, and “continuity”.

For the SI scale, the highest levels were shown for the PT dimension. The four factors – “access to help, food, and equipment”, “honesty”, “respect and empathy”, and “relatives and friends” – and three single items – “medical care”, “personal hygiene”, and “pleasant and safe atmosphere” –received the highest scores. The lowest SI scores were present for the three factors “exhaustion”, “participation”, and “spiritual and existential”.

Significant differences were present between the PR and the SI for the ID dimension and the factor “information”, where the SI was higher than the PR. No other statistically significant differences were found between PR and SI for the single items.

### Home care

The highest score on the PR scale was shown for the one factor “honesty”. The lowest PR scores were present in the MT dimension. For six of the factors the lowest PR scores were present: “symptom relief”, “exhaustion”, “information”, “participation”, “spiritual and existential”, and “continuity”.

For the SI scale, the highest scores were present in four of the factors – “honesty”, “respect and empathy”, “meaningfulness”, and “relatives and friends” – and one single item “medical care”. For the SI scale, no dimensions, factors, or single items received scores <3.00.

Significant differences were present at the MT, ID, and SC dimensions, where SI scores were higher than PR scores. For factors and single items, SI was significantly higher than PR for “symptom relief”, “information”, “meaningfulness”, “continuity”, “planning and cooperation”, and the item about medical care.

### Patients’ perceptions of care quality *across* settings

Table 3 presents adjusted mean values for the QPP-PC dimensions and single items for the PR scale when comparing patients’ perceptions of the actual care received in the four groups – hospice inpatient care, hospice day care, palliative units in nursing homes, and home care – after controlling for differences across the groups with regard to age, educational level, and number of diagnoses.

**Table 3:** Patients’ perceptions of care received *across* the four settings, by dimensions and single items

	Perceived reality (PR)										
Dimension/single item	Hospice inpatient care (HIC)		Hospice day care (HDC)		Palliative units in nursing homes (PUNH)		Home care (HC)				
	*n* = 72	Missing	*n* = 51	Missing	*n* = 30	Missing	*n* = 38	Missing			
	Adjusted mean (SE)		Adjusted mean (SE)		Adjusted mean (SE)		Adjusted mean (SE)		*F* (df, error)	*P* value*	*P* value**
Medical–technical competence	3.25 (0.10)	7	3.02 (0.10)	2	3.04 (0.13)	2	2.76 (0.13)	5	3.16 (3, 168)	0.026	
Physical–technical conditions	3.62 (0.08)	7	3.43 (0.09)	2	3.48 (0.12)	2	3.32 (0.12)	5	1.86 (3, 168)	0.138	
Identity-oriented approach	3.46 (0.06)	7	3.39 (0.07)	2	3.11 (0.09)	2	3.16 (0.09)	2	4.55 (3, 171)	0.004	HIC > PUNH: 0.013
Sociocultural atmosphere	3.45 (0.07)	8	3.34 (0.08)	2	3.25 (0.10)	3	2.95 (0.09)	4	6.45 (3, 167)	<0.001	HIC > HC: <0.001 HDC > HC: 0.008
Medical care (single item)	3.72 (0.09)	8	3.53 (0.11)	4	3.68 (0.16)	2	3.04 (0.15)	2	5.65 (3, 165)	0.001	HIC > HC: 0.001 PUNH > HC: 0.018
Personal hygiene (single item)	3.60 (0.10)	15	3.23 (0.17)	34	3.69 (0.14)	5	3.37 (0.16)	15	2.17 (3, 115)	0.095	
Pleasant and safe atmosphere (single item)	3.79 (0.05)	16	4.00 (0.06)	17	3.95 (0.07)	5	–	–	4.75 (2, 109)	0.011	HDC > HIC: 0.009

*Df* degree of freedom, *SE* standard error

The statistical level was assumed at the *P* <0.025 level

*P values refer to differences in adjusted mean between the settings, after control for differences among the groups with regard to age, education levels, and number of illnesses, measured by ANCOVA analysis

**P values refer to differences in adjusted mean between the settings, measured by Bonferroni’s test for post-hoc comparison

Patients’ perceptions of care received (PR) differed significantly between the settings for two of the four dimensions and two of the three single items on the PR scale, but none of the four dimensions or single items on the SI scale.

With regard to the PR scale, significant differences were found between patients’ perceptions of care received and settings for the two dimensions ID and SC. For the ID dimension, patients in hospice inpatient care scored the care received higher than patients in palliative units in nursing homes (*P* = 0.013). For the SC dimension, patients in hospice inpatient care and hospice day care scored higher than those in home care (*P* <0.001, *P* = 0.008). No significant differences were found between patients’ perception of care received and the independent variables “levels of education” and “number of diagnoses”. No interaction effect was statistically significant for the independent variables (settings, levels of education, and number of diagnoses) at the dimension levels.

Significant differences between patients’ perceptions of care received and settings were found for the single items about medical care and atmosphere. For the single item about medical care, hospice inpatient care and palliative units in nursing homes scored higher than home care. For atmosphere, hospice day care scored higher than hospice inpatient care. No interaction effect of the independent variables was statistically significant for any of the single items.

Results for the SI scale showed no statistically significant differences between patients’ perceptions of the importance of aspects of care and settings for the following dimensions: MT [*F*(3,167) = 0.84, *P* = 0.473], PT [*F*(3,164) = 0.79, *P* = 0.504], ID [*F*(3,170) = 0.91, *P* = 0.435], and SC [*F*(3,168) = 0.80, *P* = 0.495]. No significant differences were found between the SI dimensions and the independent variables “levels of education” and “number of diagnoses”. No interaction effect was statistically significant for any of the dimensions. Results for the SI scale showed no statistically significant differences between patients’ perceptions of the importance of aspects of care and settings for the single items with regard to medical care: [*F*(3,165) = 1.75, *P* = 0.158], personal hygiene [*F*(3,119) = 2.67, *P* = 0.051], and atmosphere [*F*(2,105) = 1.05, *P* = 0.355]. For the single item “personal hygiene”, there was a significant difference between patients’ perceptions of SI and the independent variable “levels of education” [*F*(1,119) = 9.18, *P* = 0.003]. Patients with primary or high school education scored the importance of personal hygiene higher than patients with university education (*P* = 0.003).

Interaction effects were found for the single item about medical care between settings and educational levels (*P* <0.001). Patients in hospice inpatient care and palliative units in nursing homes care who had university education scored higher, whereas patients in hospice day care and home care who had university education scored lower. Interaction effects were also found between personal hygiene and levels of education (*P* = 0.006), where patients in hospice day care, palliative units in nursing homes, and home care with primary and high school education scored higher and those with university education scored lower, whereas patients in hospice inpatient care with university level education scored higher and those with primary and high school education scored lower.

## Discussion

The participants in the present study differed across the four settings by age, education, type of diagnosis, number of diagnoses, and time in care, but did not differ with regard to their sense of coherence, psychological wellbeing, or health-related quality of life. Patients’ perception of their care *within* settings showed that high scores were present in all settings for certain care areas for both the perception of care received (PR) and the importance of care aspects (SI). For other areas, SI scored higher than PR. The results for the comparison *across* settings indicate differences in patients’ perceptions of their care across the settings, but no differences in the importance that patients perceived their care.

### Patients’ perceptions of care quality *within* settings

The results of patients’ perceptions of care quality within settings (comparing PR and SI scores) may be interpreted in two ways [27, 60]: (1) *ability of services to meet patients’ preferences for care:* care aspects with no significant difference between PR and SI (balance of the PR and SI scores, with no significant differences) could indicate that patients perceived that the care received is in line with the perceived importance. Aspects of care that received the highest scores for PR and SI (balance of *highest* PR and SI scores, with no significant differences) could imply that patients perceived they had received high-quality care on those aspects of care that were most important to them (strengths). (2) *Areas for improvement:* care areas in which SI was significantly higher than PR may be seen as areas for improvement, in that patients perceived that insufficient attention was given to the care aspect of importance to them. The possibility of the PR score being statistically higher than the SI one may also be present. This could be interpreted as the care received being perceived as better than patients’ preferences. Furthermore, the results are discussed in terms of the aspects of care in which a service’s ability to meet patients’ most important care preferences was high (balance of *highest* PR and SI scores), to illuminate the service’s strengths and discuss care areas for improvements. When interpreting the highest and lowest levels of QPP-PC scores, there is no right or wrong, and also no cut-off value. The values presented as highest (≥3.55) and lowest (<3.00) in the present study are in line with a previous study of a similar patient population [17].

The question of what levels of care are “good enough” may arise when discussing strengths and areas for improvement. Of course, from a policy-maker’s point of view, the total amount of resources and the quality of care for all patients in need of palliative care must be considered. However, healthcare systems are required to deliver high quality services and establish systems to obtain feedback from patients to be used in quality improvement work [4, 5]. In addition, the acknowledgment of the patients being the centre of care has been highlighted as important [61]. Therefore the patients’ views of what is “good enough” are important to include in the development of palliative care. In the present study, it was the patients’ perspectives that defined what was “good enough” with regard to significant differences in the PR and SI scores.

### Hospice inpatient care

The ability to meet patients’ preferences was frequently present with regard to treating patients with honesty, respect, and empathy, the planning and cooperation of services, medical care and provision of a safe and pleasant atmosphere. Studies that previously evaluated quality in inpatient hospice contexts showed similar findings [44, 62], apart from the planning and cooperation of services. Only one of these studies included the patients’ perspectives, so further studies from the patients’ perspectives are needed to confirm these findings.

Areas for improvement in the present study seemed to be related to the factor “participation”. This factor comprised questions about the opportunity to participate in the decisions that applied to medical care, nursing care, individual care planning, and choice of where to receive care. Within this factor it was the item about participation in the place of care that showed a statistically significant difference, indicating that patients prefer to be involved in decisions about place of care. There have previously been descriptions of patients’ preferences for place of care and place of death not being met [63]. Policy-makers and healthcare services responsibility for patient involvement in place of care and place of death have been highlighted by the World Health Organization (WHO) [2]. A measurement of palliative care coverage in European countries showed that the availability of health systems to meet palliative care needs of the population is still insufficient [21]. In Norway, access to hospice inpatient care is difficult as few hospice inpatient beds are available. In addition, hospice inpatient care is available only in some parts of the country. Therefore admittance to hospice inpatient care may be guided by the availability of such care and a system that prioritizes patients or patient groups, rather than by patients’ preferences.

Grande [43] has stated that it has been presumed that hospice inpatient care is a centre of excellence in palliative care, but evidence to prove whether this is true, and to provide evidence of strengths and areas for improvement, is lacking. Although more studies are needed to support the findings, the present study contributes with knowledge of hospice inpatient care about care aspects being perceived as both of high quality and an area for improvement.

### Hospice day care

The strengths seem to be related to personnel approaching patients with honesty, respect, and empathy, and that the atmosphere was perceived as pleasant and safe. Previous research supports the findings of the present study with regard to patients perceiving that hospice day care provides high-quality care for the sociocultural and identity-oriented aspects of care [37, 42, 64, 65].

Although the medical care within hospice day care received high scores, its perceived importance scored even higher. Hospice day care may be organized as a supplement to other care services, and has previously been described as having either a “medical” or a “social” focus of care [66], and that benefits are likely to be psychosocial or spiritual [37]. Even so, patients in previous studies perceived access to medical staff as important and have evaluated hospice day care as providing high-quality medical care [64, 67]. The findings in the present study – that the importance of medical care scored higher than the care received – have not been described previously. It could be an expression of the utmost importance of access to medical care.

Areas for improvement seemed to be related to the ID dimension and within this dimension the care aspect “information”. Within “information”, information about diagnosis and symptoms, prognosis, medication, and self-care showed significant values. The factor “planning and cooperation of service” is an area for improvement, and within this factor it was planning of nursing care, cooperation between personnel, and coordination of all services that the patient received that showed significant values. Parts of these findings contradict the findings of other studies in which patients perceived that the information received about living with their disease was sufficient [67] and that hospice day care was well organized [64]. However, the need for improvement in such care areas as information and continuity of care has previously been described [2, 68], although not specifically for this care context, so it needs to be explored further.

### Palliative units in nursing homes

The strengths of the palliative units in nursing homes seemed to be related to the PT dimension, including access to help, food, and equipment. In addition, the strengths related to giving honest answers to questions, care of relatives and friends, a pleasant and safe atmosphere in the wards, and sufficient care of personal hygiene. A study from a family perspective confirms that access to food (help eating) and personal hygiene (mouth care) were more frequently present for patients enrolled in specialized hospice care programs in nursing homes [69]. In addition, hospice care in nursing homes seemed to give better pain management and reduced hospitalization [70].

Areas for improvement seemed to be related to the ID dimension and, within this, receiving information related to diagnosis and prognosis had significant values. A study from nursing homes providing non-specialized palliative care confirms the need for improvement with regard to information about medical issues [33]. Further studies evaluating specialized palliative care nursing home units are needed [70], from the patients’ perspective, to confirm the findings of the present study.

### Home care

The strengths of the home-care settings seemed to be related to honest answers to patients’ questions. Honesty is recognized as an important aspect of the relationship between patients and healthcare personnel in palliative care [14]; to our knowledge, no previous study has highlighted this particular aspect of care as being fulfilled in the home-care setting. However, a study performed in advanced (specialized) homecare setting found that the identity-oriented approach of the caregiver received high scores [18].

Areas for improvement seem to be related to the ID dimension and, within this; the factor about information – specifically, information about prognosis, diagnosis, and who is the responsible nurse for the patient – was statistically significant. In addition, improvement seemed to be needed for the MT dimension, including symptom relief and medical care, and SC dimension, including meaningfulness, continuity and planning, and cooperation of care services. Previous research supports the need for improvement in similar care areas for people living at home [34, 71–73], apart from the care area meaningfulness. Help living a meaningful life have been described to include maintenance of self-worth, being with people who are important for patients, participate in meaningful activities and having hope for the future [14]. A review of Ventura et al. [73] identified spiritual needs, isolation and loss of autonomy as unmet needs in the home care settings, which partly confirms the need for improvement of the care area meaningfulness. Even if the findings in the present study need to be explored further, suggestions that can still be based upon these findings are that clinicians, leaders and policy makers should pay special attention to the areas related to providing information, symptom relief, medical care and helping patients to live a meaningful life. This important in both the everyday care of these patients, and when planning and developing further improvement initiatives and additional services.

### Patients’ perceptions of care quality *across* settings

Patients in hospice inpatient care and hospice day care were significantly younger and there were higher proportions of patients with cancer, which is in line with previous studies describing hospice inpatient characteristics [42, 74–76]. It is of interest that no significant difference was present for health-related quality of life, patients’ life orientation and ability to manage stressful situations (SOC), and psychological wellbeing. With specialized palliative care services being developed and specialized to care for patients with complex needs [1, 23], differences between settings were to be expected. However, we may not have captured all the important aspects that make up the complex needs of patients in the present study. For example we did not measure the complexity of the symptoms or need for advanced symptom control [1, 23].

The results indicate that there are differences in how patients perceived the actual care received (PR) across the different settings, although there are no differences when it comes to the perceived SI of the care aspects. Hospice inpatient care scored significantly higher than the palliative units in nursing homes, and tended to be higher than the scores for home care (*P* = 0.027) in the caregiver’s identity-oriented approach (ID dimension). Hospice inpatient and day care scored higher than home care with regard to the sociocultural atmosphere (SC dimension). Hospice inpatient care and palliative units in nursing homes scored higher than home care with regard to medical care. Specialized palliative care in dedicated hospice inpatient care and nursing homes has been shown to be beneficial compared with settings that do not specialize in palliative care [44, 74, 77] (mainly from the view of bereaved relatives) with regard to assessing multidimensional needs [74], pain control, communication, medical and nursing care [44]. One could argue that patients in hospice inpatient care and palliative units in nursing homes would be expected to score higher on aspects of care quality, because they are being admitted to services that specialize in palliative care. However, this might not be the full explanation. The evidence for the benefit of palliative care is limited [77, 78], and the results diverse. A recent study comparing decedents and relatives’ care experience, perceptions of unmet needs, and preferences in both specialized (hospital-based palliative care unit) and non-specialized settings (home care, long-term care, and hospital wards), found that none of the settings stood out as exceptional [6].

A public health approach that integrates palliative care in all levels of care with an emphasis on primary care, community and home-based care have been proposed by the WHO [79]. In Norway, the government policy is that patients should primarily be cared for at home and by community services [80]. A consequence of this policy is that patients, in the palliative phase of their illness and with more complex needs, receive care in their homes. A report [81] evaluating the effect of the policy found that home-care services do experience challenges with regard, for example, to competence to care for patients with complex needs, increased workload for nurses, challenges in availability of doctors, nurses, and other healthcare personnel, and cooperation with specialist healthcare services (hospitals). These challenges may be one reason why patients’ perceptions of care received were lower in home care compared with the other settings. In addition home care may be organized in different ways, with discrepancies in the availability of specialized services [8] such as palliative care teams, cancer nurses, and coordinators of cancer care. This could influence the perception of care quality and lead to different results in other home-care districts. For home care to be able to meet patients’ preferences for care, it is important to learn from those services that patients perceived as providing such care in an excellent way.

Patients in hospice day care scored higher than those in hospice inpatient care for the single item about a pleasant and safe atmosphere. To our knowledge, no previous study has compared these two settings for the atmosphere in the wards, although there have been studies pointing out the importance of the palliative care environment being safe and relaxing [14, 29, 82].

It is important to highlight areas of care that patients perceived to be well cared for and the setting that provided such care. Only when putting the spotlight on these areas of care and these settings is it possible to investigate further and illuminate the key ingredients in these services. It is also important to further investigate why these services excel in these areas. Other studies have shown that organizational factors, such as number of nurses [83] and whether the service delivered specialized care or not [84], could influence patients’ perceptions of the care quality. Another explanation could also be related to patients’ personal factors; for example, that gender [17], diagnosis [24, 25, 39] and symptom burden [85] could influence the perceptions of care quality. In addition, the combination of personal and organizational factors could predict how patients perceive the care quality [83]. Further studies on this are needed.

Patients’ perceptions of the SI of care aspects did not differ significantly for the dimensions and single items across the settings. Previous research has highlighted the importance of these care aspects in palliative care [14, 28, 68], and patients in the present study confirm this.

### Methodological discussion

An overall question is whether it is possible to contrast the quality of care across the four settings, with all being organized in different ways and with different patient characteristics. Even when there had been previous comparisons of perceptions of care quality across settings [6, 44], methodological issues and utility values for palliative care could arise. In the analysis differences in patient characteristics were taken into consideration and controlled for. Despite differences in care organisation across the four settings, all care services should deliver care according to the care needs of their patients. Therefore the focus of the present study was to investigate whether patients received help according to their preferences and needs, and to contrast whether their perceptions of the care quality differed across the settings, rather than comparing the settings as such. Importantly, studies comparing patient preferences in different settings are rare, and can contribute with important knowledge to improve palliative care.

### Generalizability

The strength of the present study was that it provided patients’ perspectives of the palliative care quality by including patients in a late palliative phase who were receiving palliative care. Of these participants, a high number answered the questionnaire (73 %).

There are, however, some limitations to the present study. The participants were recruited from hospice inpatient care, hospice day care, palliative units in nursing homes, and home care. Patients receive palliative care from contexts other than those included in the present study, e.g. general hospital wards, and community-based non-specialized wards in nursing homes. In addition, participants in the present study mainly had diagnoses of cancer, which could be explained by the fact that some of the settings included cared mainly for patients with cancer. The present study cannot therefore claim to have included either all relevant settings or a representative sample of the population. We have, however, provided a comprehensive description of the eligible criteria and the patient sample in each setting to clarify who was included. The interpretation of the findings must be based on the population included. A strength is that patients’ perceptions of important aspects of care did not differ across the groups of patients in the four settings included. This could mean that what is considered important to patients about their care may not be related to the settings, and therefore could be transferred to other settings. This is in line with previous studies investigating important aspects of care in a variety of settings [14, 28].

### Validity and reliability

Validated instruments were used to measure quality of palliative care [40]. For the QPP-PC the reliability in this sample was measured using Cronbach’s α, and the α values were above the desired level of 0.7 for most dimensions and factors apart from one dimension – PT (PR 0.44, SI 0.65) – and three factors – “access to help, food, and equipment” (PR 0.44, SI 0.65), “relatives and friends” (PR 0.66), and “continuity” (PR 0.53, SI 0.55). However, these dimensions and factors consisted of only three items, which may have influenced the low Cronbach’s α value [58]. The QPP-PC instrument measures both the perception of care received and the importance of the care aspects contributing to the content validity of the instrument and the study. Patients scored most of the care aspects as highly important and this importance did not differ across settings; this gives strength to the validity by showing that the QPP-PC instrument and the present study address important aspects of care.

In the present study, 54 had help filling out the questionnaire. Data collection methods may influence perceptions of care quality [86, 87] but showed contradictory results about how the results differed. We controlled whether this was the case in the present study through the use of independent-samples *t*-test, and found that patients who had help filling out the questionnaire scored significantly lower on the PR scale for one dimension – ID dimension (*P* <0.001) – and for the single item about medical care (PR) (*P* = 0.024) than patients who completed the questionnaire with no such help. More patients were interviewed in the palliative units in nursing homes and home-care districts than in hospice inpatient, indicating that the data collection method could have affected the results of the present study; this showed that patients who were hospice inpatients scored higher than those in palliative units in nursing homes on the ID dimension and higher than homecare for the item about medical care, on the PR scale. On the other hand, for the item about medical care (PR), there are still significant differences in scores between palliative units in nursing homes and home care. When testing the effect of data collection methods in each setting, results for hospice inpatient care also showed significant differences in the ID dimension (*P* = 0.012), although this was not the case for palliative units in nursing homes (*P* = 0.180), which contradicts the influence of the data collection method. Based on this, we believe that the data collection method did not bias the results, although, until our findings are supported by more studies, the results should be interpreted with caution.

When computing the QPP-PC factors and dimensions, a mean was calculated based on patients’ responses to all the items within the factor/dimension. As participants were recruited from different settings and had different diagnoses, a high proportion of “not applicable” responses for some items were expected. This was handled by calculating a mean based on the individual patient answers to the remaining items within the factor or dimension. To avoid a type 1 error the significance level was reduced to 0.025.

Grouping the participating services into settings of hospice inpatient care, hospice day care, palliative units in nursing homes, and home care, when they are organized in different ways, may be discussed. In particular, this may be a case for grouping the two hospice inpatient services. Both of these services used the term “hospice” to name and describe the services. However, they differed in their organization of care – one being delivered through community care and the other through specialist care services. Another way of grouping the setting variables could have been to group together inpatients wards delivered though community care. However, additional analyses were performed to check whether these two hospices differed significantly in the patient populations in terms of demographic data, and whether hospice care delivered through community care differed significantly from that of the palliative units in nursing homes, which was also delivered through community care. This was performed by comparing patient characteristics between the wards, using independent-samples *t*-test or Pearson’s *χ*^2^ test as appropriate. The two hospice inpatient wards did not differ significantly with regard to patient characteristics (age, education, diagnosis, multiple diagnosis, gender, contact with family, living conditions, health-related quality of life, sense of coherence, and psychological wellbeing). However, hospice care delivered through community care did differ significantly from that of the palliative units in nursing homes with regard to patient characteristics (diagnosis, multiple diagnoses, and educational level). This justified grouping together the two inpatient hospice wards in the present study’s analysis.

By performing ANCOVA, it is possible to control for bias related to differences in the groups and their effects on the results, which is a strength of the present study. Before performing the ANCOVA tests, the data were tested to see whether they met the assumptions on which the tests are built (normality distribution, homogeneity of variance, linearity, and homogeneity of regression) [58, 59]. These assumptions may influence the interpretation of the *P* values. The homogeneity of variance, tested by Levine’s test, was significant for the dimensions MT, PT, and ID, and for the single items about medical care, hygiene, and atmosphere on the PR scale. For the SI scale this was also true for the SC dimension and for single items about hygiene and atmosphere. This was probably a result of the ceiling effect [88]. To avoid misinterpretation of *P* values, their level was, as mentioned above, reduced to 0.025, in addition to choosing and performing conservative post-hoc tests for the ANCOVA analysis, as recommended by Tabachnick and Fidell [59]. The reduction of the level of *P* values was also beneficial when performing multiple analyses.

We controlled for significant differences in patient characteristics across the four settings, but we could not control for diagnosis and time in care. Patients with diagnoses other than cancer could perceive the care quality as less favourable than patients with cancer [24, 25, 39], and patients who spent longer time in care could perceive that the care quality was higher than for short-term patients [89]. This makes it difficult to assess whether differences observed across the settings have been influenced by patients’ diagnoses or time in care.

## Conclusion

Areas of strengths and improvement within each setting could guide further development of palliative care in the specific settings, by sustaining the strengths and developing specific improvement initiatives. Patients’ perceptions of the importance of care did not differ across the settings, so care areas receiving high scores on the SI scale could also be considered important when developing or improving such services. Patients’ perceptions of care received registered higher scores especially in hospice inpatient care, but also for hospice day care and palliative units in nursing homes. Further studies are needed from the patient perspective to support the findings of the present study. For further guidance of the development of palliative care, it is important to investigate why patients’ perceptions of care differ across settings and to highlight what we need to learn from those settings that receive high scores.

## Abbreviations

RRN: Registered nurse responsible for recruiting participants
ANCOVA: Analysis of covariance
HC: Home care
HDC: Hospice day care
HIC: Hospice inpatient care
ID: Identity-oriented approach dimension
MT: Medical–technical competence dimension
PR: Perceived reality
PT: Physical–technical conditions dimension
PUNH: Palliative units in nursing homes
QPP-PC: Quality from the patients’ perspective instrument specific to palliative care
RRN: Registered nurse responsible for recruiting patients
SC: Sociocultural atmosphere dimension
SI: Subjective importance
SOC: Sense of coherence
